# Supplementary material for: OGT (O-GlcNAc Transferase) Selectively Modifies Multiple Residues Unique to Lamin A
Source: Cells. 2018 May 17;7(5):44. doi: 10.3390/cells7050044 (PMC5981268; doi:10.3390/cells7050044)
Supplement: Supplementary file 1 [file cells-07-00044-s001.pdf]

SUPPLEMENTARY FIGURE 1. Peptide mapping of the lamin A/C epitopes recognized by antibody 5G4 and antibody L1293. The epitopes for lamin antibodies 5G4 and L1293 were identified by probing immobilized human prelamin A and lamin C synthetic 20-mer peptides, arrayed with 3-residue offsets (n=2; two independent peptide syntheses). (**A**) Arrays probed with lamin A/C antibody 5G4 (upper panel; +5G4 Ab), or as negative control, no primary antibody (lower panel; no 5G4). The first 566 amino acids of lamin A and C are identical, as shown in (**B**). Peptides representing the unique C-termini of lamin A and C are boxed. Amino acids relevant to antibody binding are bold; underlined residues represent the core epitope. (**C**) Aligned amino acid sequences showing that the human 5G4 epitope is conserved in mouse and rat. Identical residues are shaded black (DNAStar, Lasergene, Madison, Wisconsin). (**D**) Human lamin A/C arrays probed with antibody L1293 (upper panel; +L1293 Ab), or no primary antibody as control (lower panel; no L1293). (**E-G**) Corresponding peptides from mouse lamin A (**E**) and rat lamin A (**F**), probed with or without L1293 (left and right, respectively). Aligned amino acid sequences show the L1293 epitope is conserved in rodents (**G**). The core epitope for antibody L1293 was identified as residues 592-623, which overlap the synthetic peptide (amino acids 598-611) used as antigen by the manufacturer (Sigma-Aldrich).

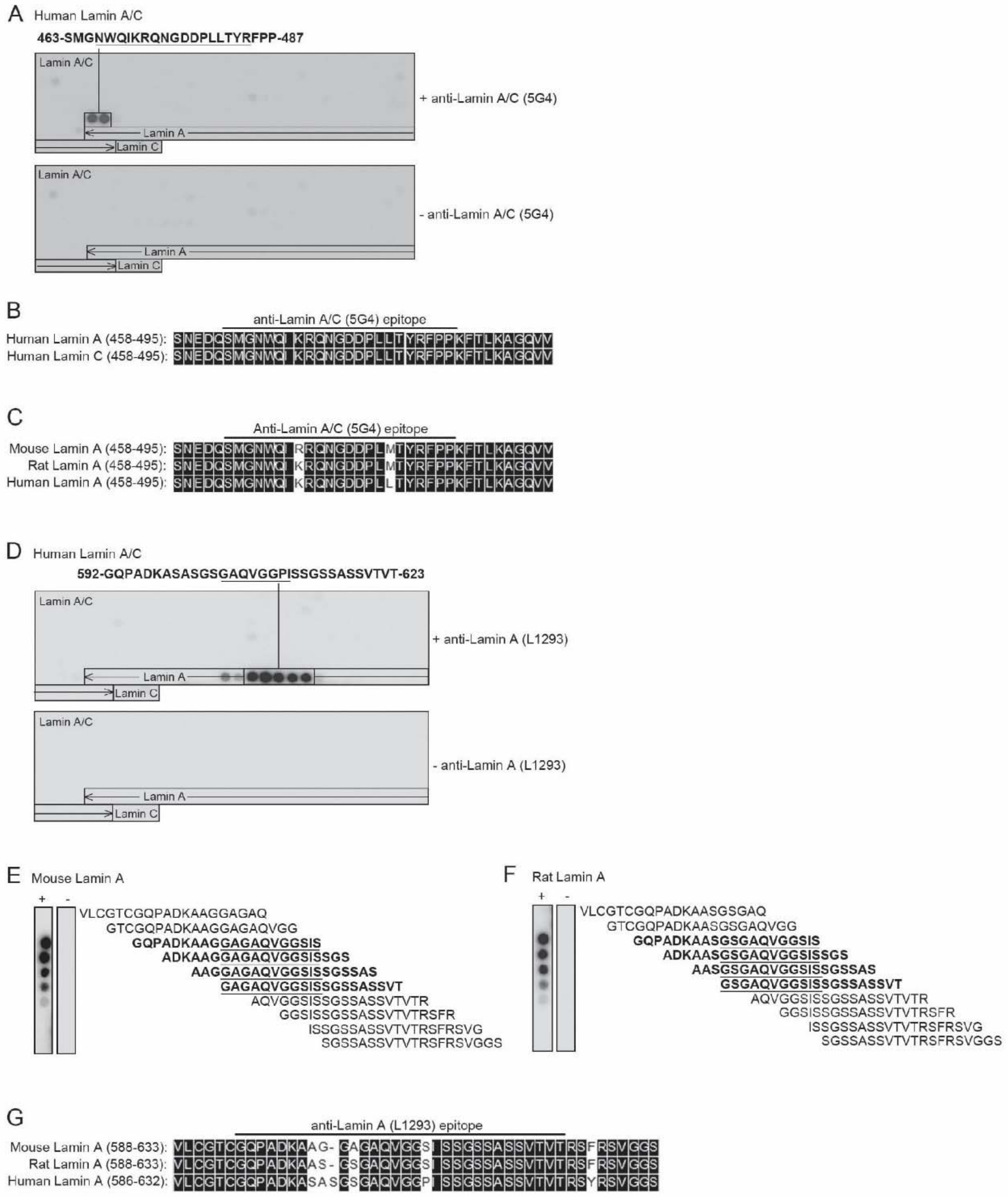

Supplementary Figure 1
